# Two-year contraceptive continuation rates among immediate postpartum implant users at a district hospital in Malawi: a prospective cohort study^☆^

**DOI:** 10.1016/j.contraception.2018.05.003

**Published:** 2018-09

**Authors:** Jennifer H. Tang, Clara Lemani, Jerome Nkambule, George Talama, Chimwemwe Banda, Webster Zgambo, Maganizo Chagomerana

**Affiliations:** a University of North Carolina at Chapel Hill, Department of Obstetrics & Gynecology, 100 Manning Drive, CB #7570, Chapel Hill, NC, 27599-7570, USA; b UNC Project-Malawi, 100 Mzimba Road, Lilongwe, Malawi; c Kasungu District Hospital, Kasungu, Malawi

**Keywords:** Postpartum, Implant, Continuation, Malawi, Africa

## Abstract

**Objective:**

To compare 2-year continuation rates in Malawian women undergoing immediate postpartum insertion of the levonorgestrel implant or etonorgestrel implant.

**Study design:**

We followed 159 women who underwent immediate postpartum levonorgestrel implant or etonorgestrel implant insertion at Kasungu District Hospital for up to 2 years.

**Results:**

We analyzed continuation data on 145 (92.4%) implant users. The 2-year continuation rates were 93.4 (95% CI 86.5–96.8) for levonorgestrel implant and 96.3 (95% CI: 76.5–99.5) for etonorgestrel implant (p=.268).

**Conclusions:**

Immediate postpartum implant insertion of both the levonorgestrel and etonorgestrel implant had high continuation rates at 2 years in Malawian women.

**Implications:**

Immediate postpartum implant insertion of both the levonorgestrel and etonorgestrel implant had continuation rates of over 90% at 2 years among our population of Malawian women. Both implants should be offered routinely to eligible and interested women prior to hospital discharge after delivery.

## 1 Introduction

Malawi has high rates of maternal mortality and unintended pregnancy [1]. Increased access to immediate postpartum long-acting reversible contraception (LARC) could decrease both. Although many countries across sub-Saharan Africa (SSA) have implemented immediate postpartum intrauterine device (IUD) insertion [2], [3], [4], [5], [6], few have implemented immediate postpartum implant insertion because it was considered to be WHO Medical Eligibility Criteria 3 (a condition where the theoretical or proven risks usually outweigh the advantages of using the method) until 2015 [7], [8]. Of the two published studies that evaluated immediate postpartum implant in SSA, neither followed women for more than 6 months after delivery [9], [10]. Therefore, our study objective was to compare 2-year continuation rates among women who received either the 5-year levonorgestrel implant or the 3-year etonorgestrel implant immediately postpartum at a district hospital in Malawi.

## 2 Material and methods

We performed a subanalysis of data collected as part of a larger implementation project of immediate postpartum LARC services at Kasungu District Hospital, a secondary-level hospital that receives referrals from all health centers in the district. Kasungu District is a rural district located in central Malawi, about 2 h north from the capital city of Lilongwe. We conducted a prospective study that recruited women who had immediate postpartum IUD or implant insertion. Because only 12 women chose a postpartum IUD, this analysis only focuses on implant insertions. Eligibility criteria for the study were women who (1) had initiated LARC within 48 h of delivery, (2) were age 18 years or older and (3) willing to return for visits every 3 months for 2 years.

As part of our implementation project, we introduced counseling about the option of immediate postpartum LARC insertion in the hospital's Antenatal Clinic, Antenatal Ward and Postnatal Ward, and trained hospital staff inserted the chosen method in interested women [11]. Our study nurses recruited women who had undergone immediate postpartum LARC insertion from the Postnatal Ward. After obtaining informed consent, eligible women completed a survey and attended follow-up visits at the hospital every 3 months until 2 years after delivery. At each follow-up visit, they completed a survey and had continuation of their implant confirmed via palpation. Our study nurses offered implant removal to participants during their scheduled study visits and any participant-initiated interim visits to ensure access to removal services, and they performed the majority of the implant removals in the study. Participants received the local equivalent of US$5 for transport reimbursement for each completed visit and remained in the study even if they discontinued their LARC method.

We used convenience sampling for our study and estimated that we could enroll up to 200 women during our 6–7 month implementation period. We double-entered data into a Microsoft Access database and exported it into Stata 15.0 for analysis. We used Fisher's Exact Test or Pearson's chi-squared test to compare baseline characteristics, Kaplan–Meier techniques to calculate rates of continuation with 95% confidence intervals (CIs), and log-rank tests to compare 2-year continuation. Both the University of North Carolina at Chapel Hill Institutional Review Board and Malawi National Health Services Research Committee approved the implementation project and the cohort study.

## 3 Results

We enrolled all 176 women who underwent postpartum LARC insertion between September 2014 and March 2015. We excluded 3 implant users less than 18 years old, 2 women with no contraceptive method recorded at enrollment, and 12 IUD users, leaving us with 159 women for baseline analysis. The characteristics of these women are presented in Table 1.

**Table 1:** Characteristics of 159 women who initiated immediate postpartum implant.

Characteristic	Levonorgestrel implant *N*=127 *n* (%)	Etonorgestrel implant *N*=32 *n* (%)	p value⁎
Age			.012⁎⁎
18–25 years	59 (46.4)	24 (75.0)	
26–34 years	56 (44.1)	6 (18.8)	
35–42 years	12 (9.5)	2 (6.2)	
Marital status			.264
Currently married	124 (97.6)	30 (93.8)	
Not currently married	3 (2.4)	2 (6.2)	
Ever attended school⁎⁎⁎			.710
Yes	117 (92.9)	29 (90.6)	
No	9 (7.1)	3 (9.4)	
Main material of roof of house			.083
Grass	83 (62.3)	26 (81.2)	
Iron sheets	44 (34.7)	6 (18.8)	
Distance to Kasungu District Hospital by walking⁎⁎⁎			.001⁎⁎
Less than 2 h	61 (53.0)	10 (32.3)	
2 h or more	36 (31.3)	6 (19.4)	
Don't know	18 (15.7)	15 (48.4)	
No. of living children			.009⁎⁎
1–2	48 (37.8)	21 (65.6)	
3–4	46 (36.2)	9 (28.1)	
5–8	33 (26.0)	2 (6.3)	
Mode of delivery⁎⁎⁎			.463
Vaginal	113 (91.1)	29 (96.7)	
Cesarean	11 (8.9)	1 (3.3)	
Pregnancy intention at time of most recent pregnancy			.103
Wanted to get pregnant then	55 (43.3)	19 (59.4)	
Did not want to get pregnant then	72 (56.7)	13 (40.6)	
Number additional children wanted in future⁎⁎⁎			<.001⁎⁎
0	49 (38.9)	3 (9.4)	
1	51 (40.5)	12 (37.5)	
2–4	23 (18.2)	15 (46.9)	
Don't know	3 (2.4)	2 (6.2)	
Ever used a family planning method			.021⁎⁎
Yes	94 (74.0)	17 (53.1)	
No	33 (26.0)	15 (46.9)	
Has friends who have used the implant⁎⁎⁎			.164
Yes	93 (73.8)	21 (65.6)	
No	33 (26.2)	11 (34.4)	
Partner aware that she is using implant			.746
Yes	91 (71.7)	22 (68.8)	
No/Don't know	36 (28.3)	10 (31.2)	

⁎ Calculated using Fisher's Exact Test or Pearson's chi-squared test.

⁎⁎ p value < .05.

⁎⁎⁎ Missing data or no response.

Fourteen women (8.8%) never returned for a follow-up visit. Therefore, we analyzed continuation rates for 145 (91.2%) women: 117 (92.1%) of the 127 levonorgestrel implant users and 28 (87.5%) of the 32 etonorgestrel implant users. We calculated 2-year continuation rates of 93.4 (95% CI 86.5–96.8) for the levonorgestrel implant and 96.3 (95% CI: 76.5–99.5) for the etonorgestrel implant (p=.625). In a sensitivity analysis in which participants were considered discontinuers when lost-to-follow-up, we found 2-year continuation rates of 78.4 (95% CI 69.7–84.9) for the levonorgestrel implant and 82.1 (95% CI: 62.3–92.2) for the etonorgestrel implant (p=.703).

The one etonorgestrel implant discontinuer and one of the seven levonorgestrel implant discontinuers requested implant removal because they underwent sterilization. Among the six remaining levonorgestrel implant discontinuers, three requested implant removal because of bleeding complaints, one because of cough, one because of palpitations and one because she wished to conceive (her infant died at 6 months from surgical complications).

## 4 Discussion

Less than 10% of our immediate postpartum implant users discontinued their method at 2 years. This proportion is similar to the 1-year discontinuation rates for the general population in Malawi, which is 8.6% for implants [1]. Our discontinuation rate may be low because our population only included postpartum women, who may have been motivated to prevent another pregnancy or achieve adequate birth spacing before their next pregnancy. Our immediate postpartum LARC rate of 3.8% is similar to the 2.3%–5.8% uptake found in six other countries that were implementing only immediate postpartum IUD [4] but not as high as the 41% found in one study in Nigeria, which was performed only in private hospitals [3]. However, our immediate postpartum implant continuation rates are comparable to those found in a randomized controlled trial in Uganda, in which 97% of women randomized to immediate postpartum insertion were using the implant at the 6-month follow-up visit [10]. Our study results emphasize the need to expand access to immediate postpartum implant insertion now that the World Health Organization has upgraded it from Category 3 to 2 (a condition where the advantages of using the method generally outweigh the theoretical or proven risks) [8].
